# Flexible Memory Application of Nanoscale Ti–Ge–Te Thin Film as Information Storage Medium With Excellent Thermal Stability, Low Resistance Drift and Superior Bending Characteristic

**DOI:** 10.1002/advs.202507859

**Published:** 2025-08-04

**Authors:** Han Gu, Weihua Wu, Xiaochen Zhou, Pei Zhang, Bowen Fu, Jiwei Zhai, Sannian Song, Zhitang Song

**Affiliations:** ^1^ School of Mathematics and Physics Jiangsu University of Technology Changzhou 213001 China; ^2^ National Laboratory of Solid State Microstructures Nanjing University Nanjing 210093 China; ^3^ Shanghai Key Laboratory for R&D and Application of Metallic Functional Materials School of Materials Science & Engineering Tongji University Shanghai 201804 China; ^4^ State Key Laboratory of Functional Materials for Informatics Shanghai Institute of Microsystem and Information Technology Chinese Academy of Sciences Shanghai 200050 China

**Keywords:** crystallization property, flexible material, mechanical performance, phase change mechanism, Ti–Ge–Te thin film

## Abstract

The flexible Ti–Ge–Te phase change material was proposed and fabricated by magnetron cosputtering method. The impact of titanium dopant and mechanical bending on the thermal stability, electrical resistance, surface morphology, microstructure, and crystallization mechanism of GeTe thin film are investigated systematically. With the incorporation of appropriate titanium dopant, the crystallization process and grain size of GeTe material can be hindered. Meanwhile, the thermal stability, surface morphology, and crystal structure have not been changed obviously when the bending times reaching 10^6^, demonstrating the distinguished mechanical bending performance. The phase change memory devices with Ti‐doped GeTe were prepared based on flexible polyimide substrates, and the electronical properties are evaluated. The consequences show that the phase change memory can still exhibit the negative resistance phenomenon and complete the erase/write operation after bending 10^6^ cycles. The density functional theory calculations of band structure illustrate that titanium dopant can convert the indirect band gap of GeTe material to the direct type. The formation energy and charge density difference indicate the massive electron cloud agglomerate between the Ti and Te atoms, deducing that the foreign Ti may occupy the position of Ge and form the covalent bonds with Te.

## Graphical abstract

Nanoscale Ti–Ge–Te films were fabricated via magnetron co‐sputtering, exhibiting the exceptional thermal stability, low resistance drift, and superior mechanical flexibility. Density functional theory reveals Ti doping can convert GeTe to a direct bandgap semiconductor and enhance covalent Ti–Te bonding. Flexible phase‐change memory devices demonstrate the reliable switching at electrical pulse with 5 ns, showcasing the potential for wearable electronics applications.

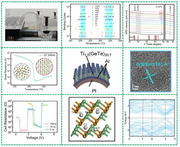

## 1 Introduction

Owing to the emergence of wearable electronic terminals and digital information acquisition technology based on natural motion sensing, the Internet of Things (IoT) and artificial intelligence (AI) are undergoing an unprecedented revolution^[^ ^1^, ^2^, ^3^ ^]^ However, these technologies highly rely on flexible substrates coupled with various form factors and surfaces.^[^ ^4^ ^]^ The current memory manufacturing technology faces the enormous challenges to overcome the limited flexibility of silicon wafers, the complexity of transfer process, and the low thermal conductivity to integrate conventional silicon substrate memories into flexible electronics.^[^ ^5^, ^6^ ^]^ It has been reported that fabrication of flexible memories usually be performed below 250 °C, and the flexible devices often requires higher power and operating voltages. Phase change memory (PCM) has been regarded as the main competitor of the next generation nonvolatile memory, which has faster on‐chip speed, larger storage window, long write/erase persistence, great anti‐radiation properties, and low resistance variability.^[^ ^7^, ^8^, ^9^ ^]^ It has broad application prospects in various fields, including consumer electronics and automotive electronics, even aerospace electronics. Therefore, designing and achieving high‐performance flexible storage material and devices have drawn widespread attention for wearable electronic terminals.^[^ ^4^ ^]^

**Figure 1:**
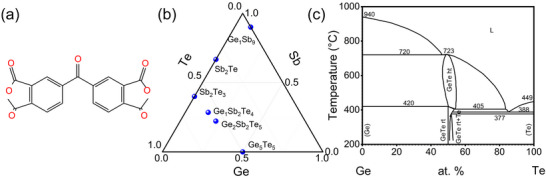
a) Chemical structure of PI; b) Ternary phase diagram of Ge–Sb–Te alloy; c) phase diagram of binary Ge–Te condensed system. The abbreviation of rt, ht, and L represent room temperature, high temperature and liquid, respectively.

Polyimides (PI) are widely used in microelectronics, sensors, biomedical and aerospace applications due to their excellent combination of properties such as high thermal stability, mechanical strength, chemical resistance, dielectric properties and biocompatibility.^[^ ^10^ ^]^ **Figure** **1**a shows the chemical structure of PI.^[^ ^11^, ^12^ ^]^ Among the various phase change materials, the pseudo‐binary tie from GeTe to Sb_2_Te_3_ has obtained the great popularity, as shown in Figure 1b, especially GeTe possesses high crystallization temperature and fast switching speed. Figure 1c illustrates the phase diagram of binary Ge–Te condensed system. At around 723 °C, GeTe with a stoichiometric ratio of 1:1 melts consistently into liquid‐phase GeTe, having both a room‐temperature and high‐temperature crystalline phase. The crystalline phase at room‐temperature is the triclinic phase created by the deformation of the rock‐salt structure along the body diagonal, while the crystalline phase at high‐temperature is the cubic direction of the rock‐salt structure. The extended service life of Ge–Te material ascribes to the stable physical and chemical characteristics, which can avoid phase separation during the cyclic erasing/writing. The large resistance ratio between the amorphous and polycrystalline state not only can ensure the reliability of data readout in traditional binary data storage, but also can provide a critical foundation and potential for device applications in brain‐inspired computing (neuromorphic computing), as it can create the necessary conditions for achieving the wide dynamic resistance range required to simulate synaptic weight changes. ^[^ ^13^, ^14^ ^]^ In addition, the amorphous state becomes more resilient to thermal fluctuations and crosstalk at the higher crystallization temperature, which is advantageous for the GeTe application in the high‐density storage.^[^ ^15^, ^16^ ^]^ However, the larger grain size will result in the poorer stress buffering, defect absorption capacity, uniformity and repeatability of the crystalline‐amorphous process in thin films, and a shorter cycle life for devices.^[^ ^17^ ^]^ GeTe possesses 10% intrinsic Ge vacancies, which provides the possibility of modification by doping with trace elements.^[^ ^18^ ^]^ In previous studies, the modification of GeTe materials by doping with N,^[^ ^19^ ^]^ C,^[^ ^20^ ^]^ Cr,^[^ ^21^ ^]^ Cu,^[^ ^22^ ^]^ Ag,^[^ ^23^ ^]^ Mo^[^ ^24^ ^]^ and other elements has been reported. During the device preparation process, PCM often utilizes titanium or titanium nitride as electrical electrode or adhesion layer. In a few studies, the microstructure of titanium diffusion, *RESET* current reduction, or titanium/titanium nitride diffusion thermal resistance in PCM has been analyzed using high‐resolution transmission electron microscopy. The recording medium is repeatedly subjected to high thermal stresses and therefore requires appropriate mechanical properties to ensure the reliability of the PCM, mainly in respect of fatigue properties and mechanical stability.^[^ ^25^ ^]^ Thus, we choose GeTe as matrix material and Ti element as dopant to form the nanocomposite thin film, hoping to obtain the high‐performance flexible PCM in electrical, thermal and mechanical properties.

In this work, we synthesized GeTe and Ti‐doped GeTe films on polyimide substrates, and the effect of mechanical bending on the surface, microstructure, crystalline phase structure, and thermal stability were investigated in detail. The electronical performance of PCM devices based on Ti‐doping GeTe film was evaluated. Also, the impact of Ti on the modulation of GeTe band structure and partial density of states (PDOS) based on density functional theory (DFT) were examined, revealing the improvement mechanism of Ti doping on GeTe material.

## 2 Results and Discussion

**Figure 2:**
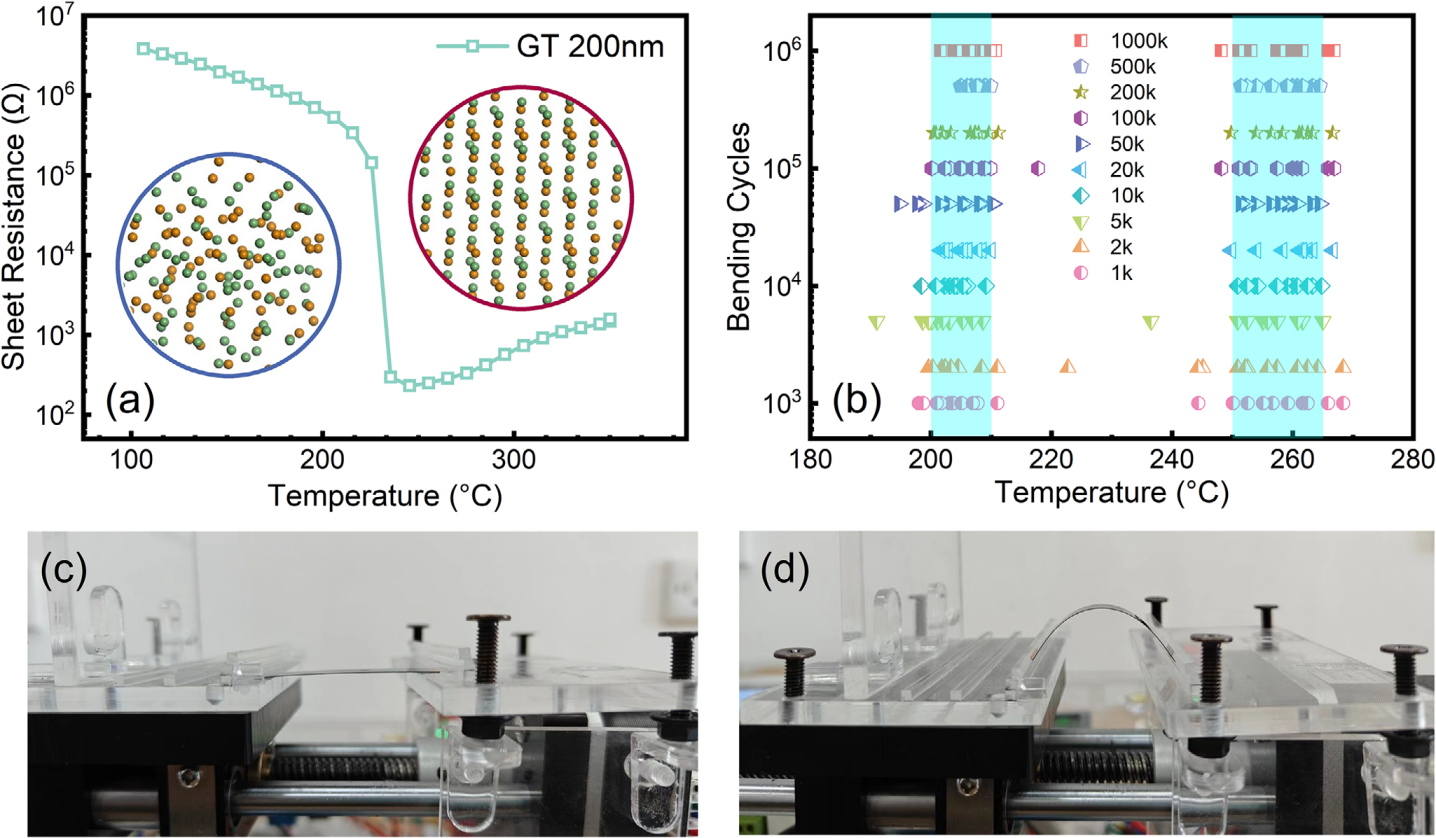
a) *R–T* curve of GT film. The insets show the GT cells in amorphous (bottom‐left) and crystalline (top‐right) states. b) *T_c_* values of GT and TiGT films under the circumstance of different bending cycles. The flat c) and bending d) state of samples.

An *in‐situ* resistance‐temperature system was used to investigate the relationship between the bending cycles and the annealing temperature. **Figure** **2**a shows *R–T*curves of GT film, exhibiting the obvious phenomenon with resistance mutation. Initially, the film has a high resistance value, which indicates that the film is in an amorphous state.^[^ ^26^, ^27^ ^]^ The resistance value of GT film consistently drops as the temperature rises, displaying the characteristics of a semiconductor.^[^ ^28^ ^]^ The temperature node at which the resistance decreases sharply during heating is defined as crystallization temperature (*T_c_*). When the GT film reaches *T_c_*, the resistance drops rapidly from 10^5^ to 10^2^ Ω, which may be due to the great change in carrier concentration or electron mobility during the crystallization from the disordered amorphous state to the ordered crystalline state. The crystalline and amorphous models in the inset were constructed by performing molecular dynamics calculations on the cells using the Kinetics Calculation task in the Forcite Calculation Module of the Materials Studio software. As shown in Figure 2a, the ratio of amorphous resistance (*R_a_*) to crystalline resistance (*R_c_*) of GT film exceeds three orders of magnitude. The larger resistance ratio of thin film means the high signal‐to‐noise ratio of PCM device, which can avoid the data misreading and ensure the reading accuracy.^[^ ^29^ ^]^

To explore the impact of Ti dopant and mechanical bending on the thermal property of GT thin film, we designed and selected a few of different bending numbers. The scatter plots of the bending cycles versus crystallization temperature are displayed in Figure 2b. To make the measurement more accurate and prove the repeatability of results, 11 samples were chosen and tested with equal precision. From Figure 2b, it can be found that the *T_c_* value of GT films ranges from 200 to 210 °C, while TiGT scopes from 250 to 265 °C. Obviously, the *T_c_* of GT films increase significantly after doping Ti element, indicating the great improvement in thermal stability.^[^ ^30^ ^]^ Besides, the *T_c_* s of GT and TiGT films with PI substrate can maintain within a certain temperature range after bending different cycles, suggesting that the superior mechanical characteristics of GT and TiGT films. Figure 2c,d present the bending instrument with the samples in flat and flexural states, respectively.

**Figure 3:**
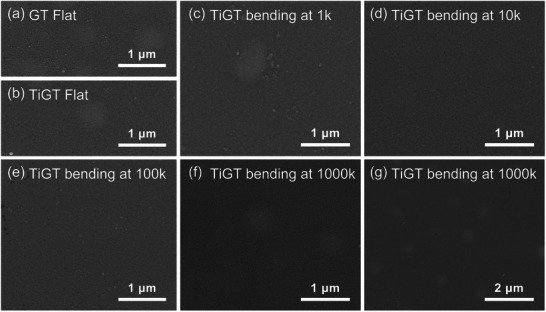
SEM images of flat films without bending: a) GT, b) TiGT. Surface of TiGT films under the circumstance of different bending cycles: c) 1k, d) 10k, e) 100k, f) 1000k. g) Zoom out image of TiGT film after bending 1000k.

To ascertain the influence of bending cycles on the surface morphology, the GT and TiGT films deposited on PI substrate were observed by SEM. **Figure** **3**a,b depict the surface morphology of GT and TiGT films in flat state, exhibiting the smooth surface. Figure 3c‐f display the SEM pictures of TiGT films with various bending cycles, showing no visible cracks or cavities. Also, the zoom out image of TiGT films reveals that there are no obvious flaws, as shown in Figure 3g. The observation from SEM indicates that the mechanical bending has no negative impact on the surface of thin films, further demonstrating the superior flexibility of TiGT film with PI substrate.

**Figure 4:**
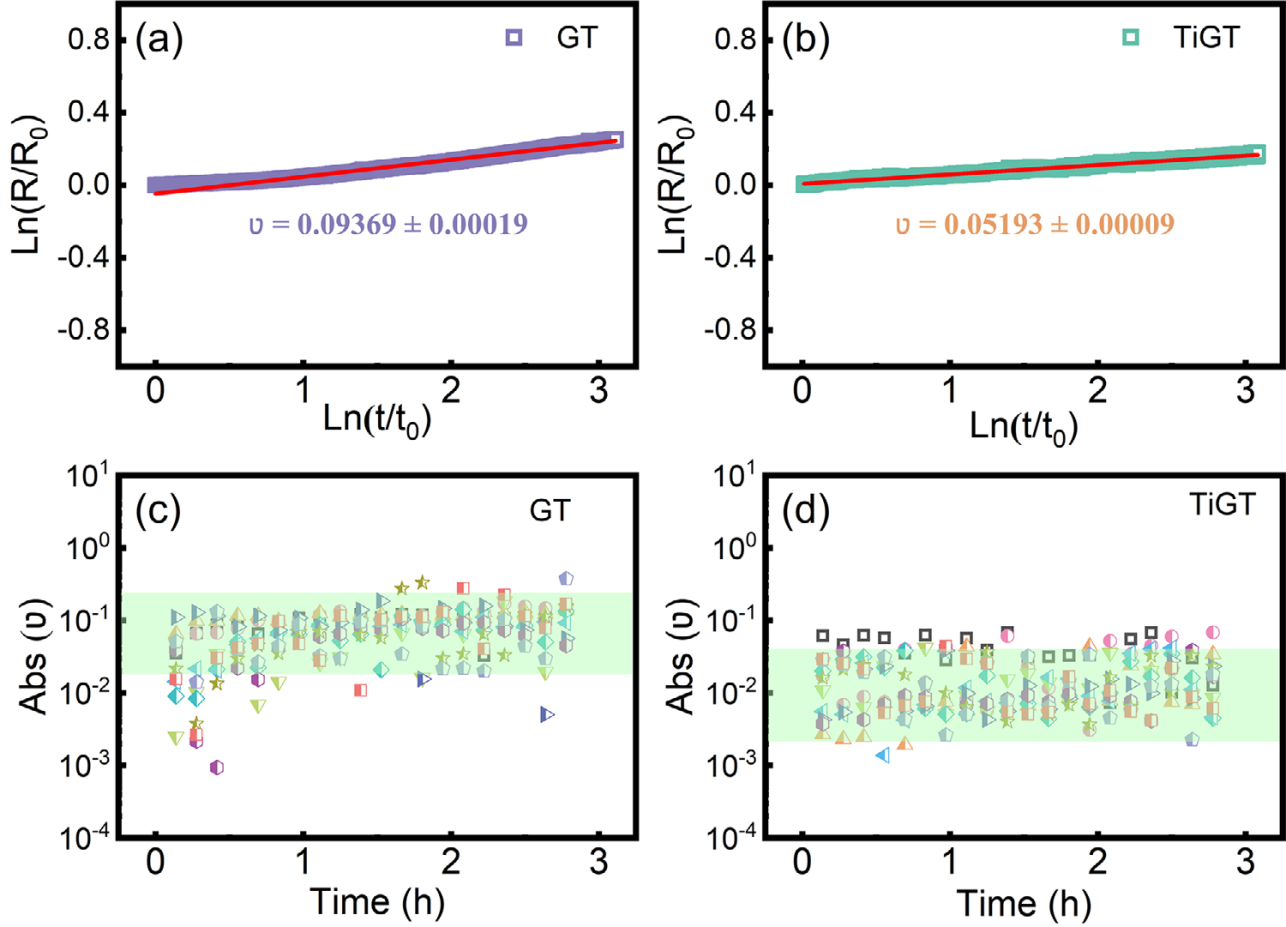
Resistance drift curves of GT a) and TiGT b) films without bending. Absolute values of the resistance drift coefficients as a function of time corresponding to GT c) and TiGT d) films.

The amorphous phase change materials exhibit significant spontaneous structural relaxation, which is known as the resistive drift phenomenon. The resistance drift is one of the most important factors influencing PCM applicability, which can reduce the reading accuracy after a large number of erase/write operations.^[^ ^31^, ^32^ ^]^ As shown in **Figure** **4**a,b, the resistance drift curves of flat GT and TiGT films are fitted by the following equation^[^ ^33^, ^34^ ^]^

(1) $$R_{t}=R_{0}{\left(\frac{t}{t_{0}}\right)}^{\nu }$$

where ν is the resistance drift coefficient, *R_t_* and *R*_0_ represent the initial and final resistance value measured at *t* and *t_0_*, respectively. In contrast to the flat GT film, the resistance coefficient |ν| of TiGT decreases from 0.09369 to 0.05193. By restricting the structural relaxation of phase change material and lowering the kinetics of the supercooled metallic liquid, this nanoscale wall can lower |ν| and increases the stability of the amorphous resistance.^[^ ^35^ ^]^ To clearly examine the dependence of |ν| on time, we divided the entire drift curve into consecutive segments. This was derived by fitting the segmented resistance‐temperature data (each subset of ∼500 s), the |ν| represents the speed of microstructure collapses over time.^[^ ^36^ ^]^ As shown in Figure 4 c,d, the numerical values |ν| of GT films after bending different numbers range from 10^−1^ to 10^−2^, while TiGT films are in the range of 10^−2^ to 10^−3^. This may owe to the creation of new bonds and the inhibition of the structural relaxation of the amorphous phase after Ti doping,^[^ ^37^ ^]^ which improves the stability of the amorphous and the accuracy of the data identification, then preventing the occurrence the chaotic phenomena. It is noteworthy that the absolute values |ν| of GT and TiGT films do not depend on the bending cycles, indicating that the structure of GT and TiGT films based on PI has not been influenced by mechanical bending.

**Figure 5:**
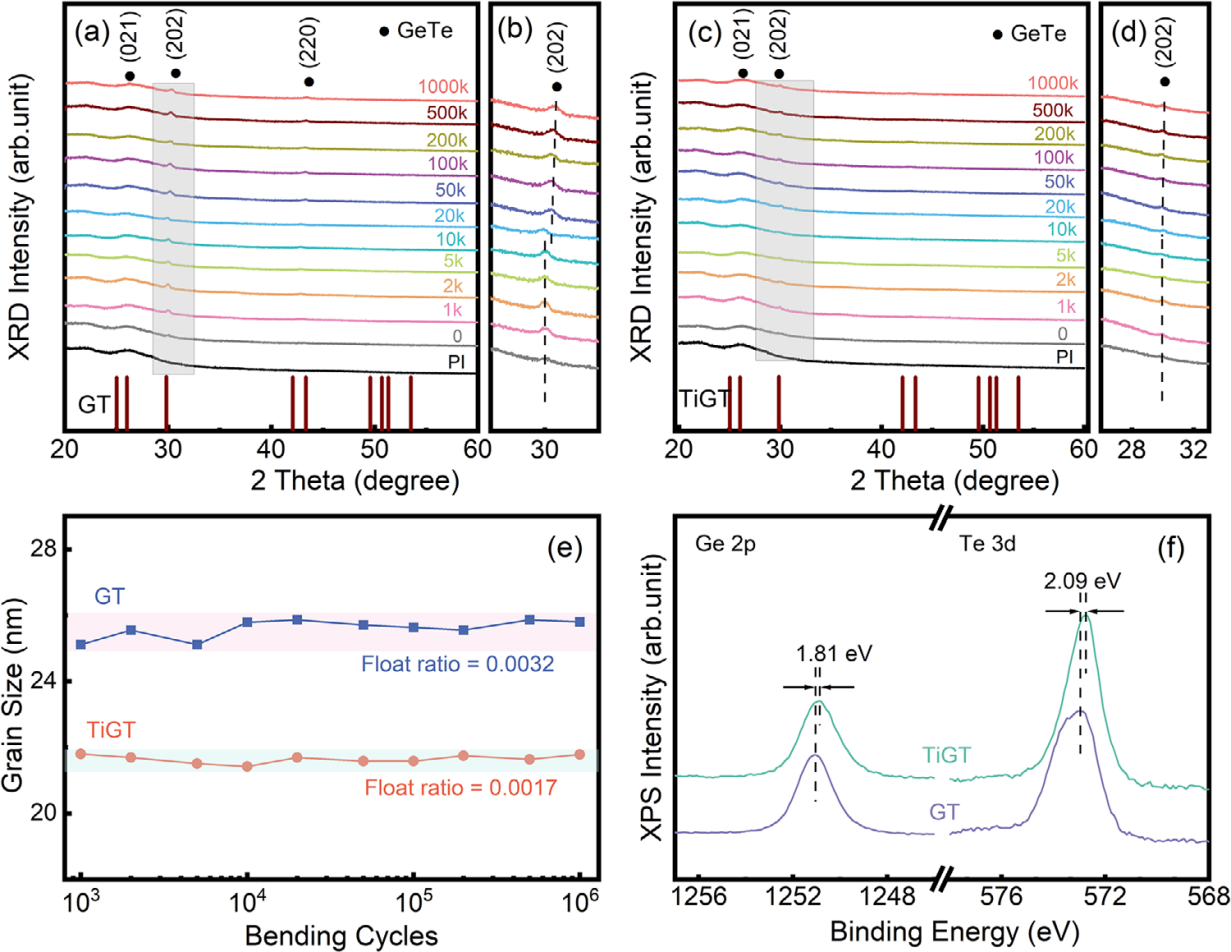
XRD patterns of GT a) and TiGT c) films after different bending number; b,d) Magnification of the diffraction peak (202) in (a) and (c), respectively; e) plots of the grain size and bending cycles; f) XPS patterns of Ge 2p and Te 3d of GT and TiGT materials.

To further investigate the effect of bending and Ti doping on the crystallization process of GeTe thin films, the phase structure of GT and TiGT thin films with different bending numbers was analyzed by XRD, which are shown in **Figure** **5**a–d. In order to avoid some influence on the lattice information by possible defects during the bending process, the prepared GT and TiGT films were annealed at 300 °C for 10 min. Then the mechanical bending was performed for a corresponding number of times. In Figure 5a, for GT films with different bending cycles, the diffraction peaks appearing at 2*θ* = 30.1 and 42.9 ° are identified as (202) and (220) Bragg reflections of GeTe, respectively. Based on the positions of diffraction peaks and standard PDF card, the annealed GT films are determined to hexagonal (HEX) crystal structure, which has superior structural stability, packing density and symmetry. In Figure 5b, the TiGT films exhibit the only diffraction peaks of GeTe (202) with HEX structure at 2*θ* = 30.1°. Compared to the diffraction peak exhibited in pure GT films, the intensity of the diffraction peak (202) is reduced and the diffraction peak (220) disappears in the TiGT films. The weaking of intensity of diffraction peak (202) indicates that Ti doing atoms can suppress the phase transition process and refine the grain growth, improving the thermal stability of GT film. The disappearance of diffraction peak (220) may be due to a change in the preferred orientation of the grains after Ti doping. Furthermore, no other crystalline phase exists except the single GeTe phase, indicating that Ti doping does not change the crystalline phase of GT film,^[^ ^27^, ^38^ ^]^ and the single crystalline phase can effectively prevent phase separation, which is conducive to improve the durability of PCM devices.^[^ ^39^ ^]^

Figure 5b,d shows the magnified view of diffraction peak (202) in GT and TiGT films, respectively. In Figure 5b, the diffraction peak (202) produces a regular slight shift when the number of bending reaches 50–100k, which may be due to the change of average strain level of the lattice.^[^ ^40^ ^]^ There is also a slight increase in peak width during bending, which may be caused by the increase in nonuniform deformation of the lattice. The width of the diffraction peak can also reflect the degree of grain refinement, the wider the diffraction peak, the smaller the grain size. It can be observed by the auxiliary line in Figure 5d that the shift of diffraction peak (202) is significantly smaller and the peak width is obviously wider compared to GT film, which may due to the fact that Ti dopant can suppress the lattice distortion and refine the grain size.^[^ ^41^ ^]^ For the diffraction peak GeTe (202), the lattice parameter of c‐GT was deduced to be 10.662 Å in the XRD pattern for the undoped GT films, whereas the lattice parameter of c‐TiGT in the TiGT films increased slightly to 10.668 Å. The increase in the lattice strain is directly attributed to the decrease in the intrinsic Ge vacancies inside the GeTe sublattice because of the presence of excess homopolar anions.^[^ ^42^ ^]^ The substitution of Ge atoms by Ti also becomes a reduction of intrinsic Ge vacancies inside the GeTe sublattice, which results in the relaxation of cubic structure and leads to an increase in GeTe lattice parameter.

The grain size of GT and TiGT with different bending cycles can be calculated by the Scherrer formula^[^ ^43^, ^44^ ^]^

(2) $$D_{hkl}=0.943\frac{\lambda }{\beta cos\theta }$$

where *D_hkl_*, *λ*, *β*, *θ* represent grain size, the wavelength of X‐rays (0.514 nm), the full‐width at‐half‐maximum (FWHM), and the diffraction angle. The calculated results are shown in Figure 5e, it can be founded that the grain sizes of GT films range from 25.11 to 25.87 nm, whereas TiGT films oscillate between 21.42 and 21.80 nm, exhibiting the more concentrated distribution and greater stability. Therefore, XRD results further illustrate that excellent mechanical performance of TiGT films.

To analyze the precision of experimental results, the relevant floating ratio of grain size was calculated by type A uncertainty equation

(3) $$\mu_{A}\left(D\right)=\sqrt{\frac{1}{n\left(n-1\right)}\sum_{i=1}^{n}{\left(D_{i}-\bar{D}\right)}^{2}}$$

where *n* denotes the number of samples, *D_i_* denotes the *i* ^th^ measurement of grain size, and $\bar{D}$ denotes the average of grain size. Calculations prove that the floating ratio of the grain size of GeTe (202) of GT films is 0.0032, while TiGT films is as small as 0.0017, which is about half of GT's value. Thus, doping Ti can effectively inhibit the crystallization process and limit the grain size. Smaller grain size can cause more carrier scattering and larger grain boundaries, improving the crystalline resistance of phase change materials and reducing the programming current of PCM device.^[^ ^45^ ^]^

To explore the effect of Ti dopant on bonding state of GT and TiGT thin films, high‐resolution XPS spectroscopy were utilized to characterized the binding energy of constituent elements. As shown in Figure 5f, two peaks of Ge 2p and Te 3d of GeTe film are located at the binding energies of 1251.04 and 572.95 eV, respectively. After doping with Ti, the two peaks of Ge 2p and Te 3d shift toward the lower binding energies by 1.81 and 2.09 eV, respectively. The negative shifts of binding energies of Ge and Te may stem from the fact that the electronegativity of Ti (1.54) is smaller than that of Ge (2.01) and Te (2.10).^[^ ^46^, ^47^ ^]^ This may due to some of Te atoms in Ge‐Te bond are replaced by Ti atoms to form Ti–Ge and Ti–Te bonds, forming the amorphous regions surrounding the grains and causing the inhibition of grain growth.^[^ ^47^ ^]^ The XPS analysis is consistent with the above XRD results. The tendency of Ti dopant bonding with Ge and Te elements will be further discussed in the subsequent first‐principles calculations.

**Figure 6:**
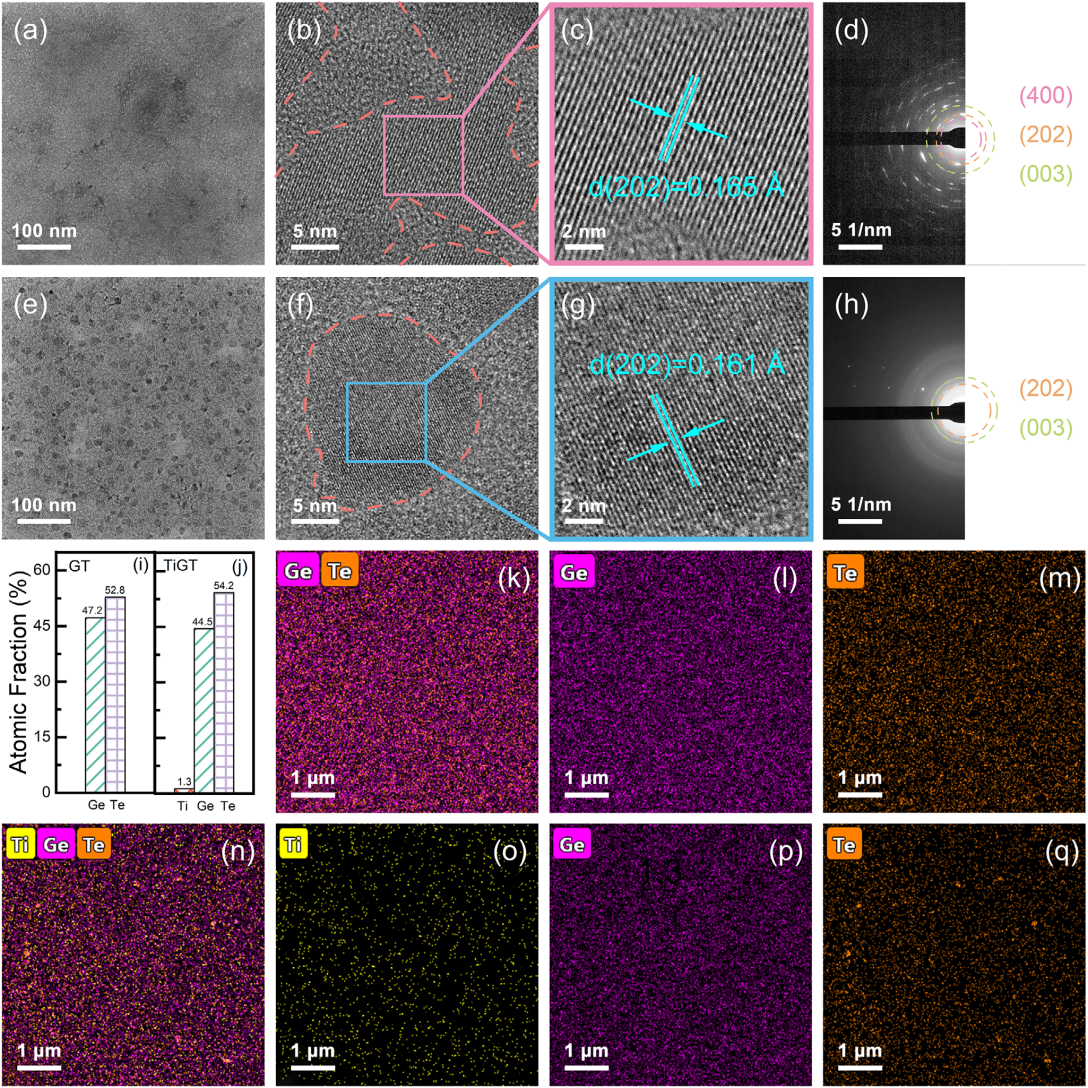
BFTEM images with corresponding HRTEM and SAED patterns for GT a–d) and TiGT e–h) films, respectively. The irregular red dashed lines in (b) and (f) indicate the demarcation between the amorphous structure and the lattice fringing, and the colored solid lines correspond to the magnified lattice stripes in (c) and (g), respectively. Statistical chart of atomic fraction of elements in the GT i) and TiGT j) films. Energy dispersive X‐ray spectroscopy (EDX) mappings of GT k–m) and TiGT n–q) films.

The crystal morphology and microstructure of GT and TiGT thin films were further characterized by TEM. Before TEM test, the test samples were annealed at 300 °C for 8 min to obtain films with great crystallinity. **Figure** **6**a–d and 6e–h indicate the corresponding bright‐field transmission electron microscopy (BFTEM), high‐resolution transmission electron microscopy (HRTEM), and selected‐area electron diffraction (SAED) maps of GT and TiGT films, respectively. From Figure 6a and 6e, it can be observed that both the samples crystallize well with uniform grain distribution. After doping Ti element, the grain size decreases obviously, verifying that Ti plays a role in inhibiting crystallization as mentioned in XRD.^[^ ^41^ ^]^ With the grain size reducing, the probability of grain boundary diffusion or sliding effect also decreases. The residual stress between the film and interface is positively correlated with the grain size, the reduction of residual stress can lead to a further decrease in the root‐mean‐square surface roughness of the film.^[^ ^35^, ^48^ ^]^ This means that the smaller grain size, the more reliable of PCM device.^[^ ^49^ ^]^

As shown in Figure 6b and 6f, the polycrystalline films are composed of nanograins with different crystallographic orientations, which are surrounded by amorphous regions.^[^ ^50^ ^]^ The irregular red dotted box shows the amorphous structure in the film, and the square frame lines in pink and blue correspond to the magnified lattice stripes in (c) and (g), respectively, which were determined to be the GeTe (202) phase by checking the standard PDF card carefully. Figure 6d,h demonstrates the polycrystalline structural features of GT and TiGT films, respectively. The continuity of diffraction rings was observed by selecting the same region for evaluating the change in grain size. The continuity of diffraction rings is closely correlated with the gain size. Comparing Figure 6d with Figure 6h, it can be found that the continuity of polycrystalline diffraction rings of TiGT films is significantly better than that of pure GT, which suggests that TiGT film contain smaller grain sizes and verifies the crystallization inhibition effect of Ti dopant. The elemental distributions of GT and TiGT films that were studied by TEM with EDS mapping. The percentages of each element of GT and TiGT films are demonstrated in Figure 6i,j, the content of Ti is only 1.3%. As illustrated in Figure 6k‐m and n–q, it can be observed that the atomic distribution of GT and TiGT films is relatively uniform. The relatively low spatial distribution of Ti elements compared to other elements may be attributed to the low doping of Ti. The observations of TEM further indicate that Ti dopants can inhibit the grain growth and refine the grain size, which has a positive influence on improving the thermal stability and crystallization resistance of GT thin film.

**Figure 7:**
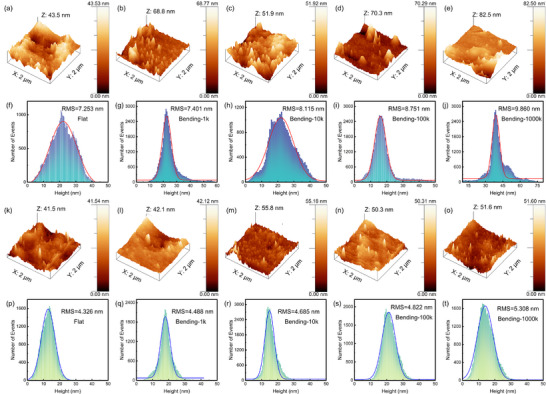
AFM images of GT a–e) and TiGT k–o) films after different bending cycles. (f–j) and (p–t) are the corresponding height histograms of (a–e) and (k–o), respectively.

During the reversible phase transition process, the stresses generated by internal structural changes will affect the interfacial quality between the phase change layer and electrode.^[^ ^51^ ^]^ Therefore, the smoothness of film surface is one of the key factors influencing the reliability of device. The surface morphology of GT and TiGT films with different bending times was observed by AFM, which is shown in **Figure** **7**. Root mean square (RMS) of microscopic peaks and valleys can be commonly used to quantitatively evaluate the surface roughness of thin films. As shown in Figure 7a,k, the RMS values of GT and TiGT films are 7.253 and 4.326 nm, respectively. The RMS of TiGT films is significantly lower than that of GT, ascribing to the crystallization inhibition after doping Ti. The smoother surface topography can improve the contact quality between the film and electrodes, contributing to increase the reliability and life of PCM device.^[^ ^52^, ^53^ ^]^ Figure 7b–e and l–o represent the surface topography of GT and TiGT films under different bending cycles, respectively. When GT and TiGT samples bending at various cycles between 0 and 1000 k, the film surface exhibits the consistent distribution without any noticeable creases, fissures or flaws, inferring the superior mechanical performance.

The surface height histograms of GT and TiGT films corresponding to various bending numbers are shown in Figure 7f–j and p–t, respectively. Fitting the height histograms, it can be found that all curves conform to Gauss normal distribution, which indicates that the grains distribute uniformly on the film surface with no obvious bumps or depressions. With the increasing of bending number, the RMS value increases slightly, which is consistent with the trend of grain size expressed in Figure 5e. Combing with the conclusion in Figure 3, mechanical bending has little effect on the film surface morphology, which further proves that TiGT films have superior flexibility characteristic.

**Figure 8:**
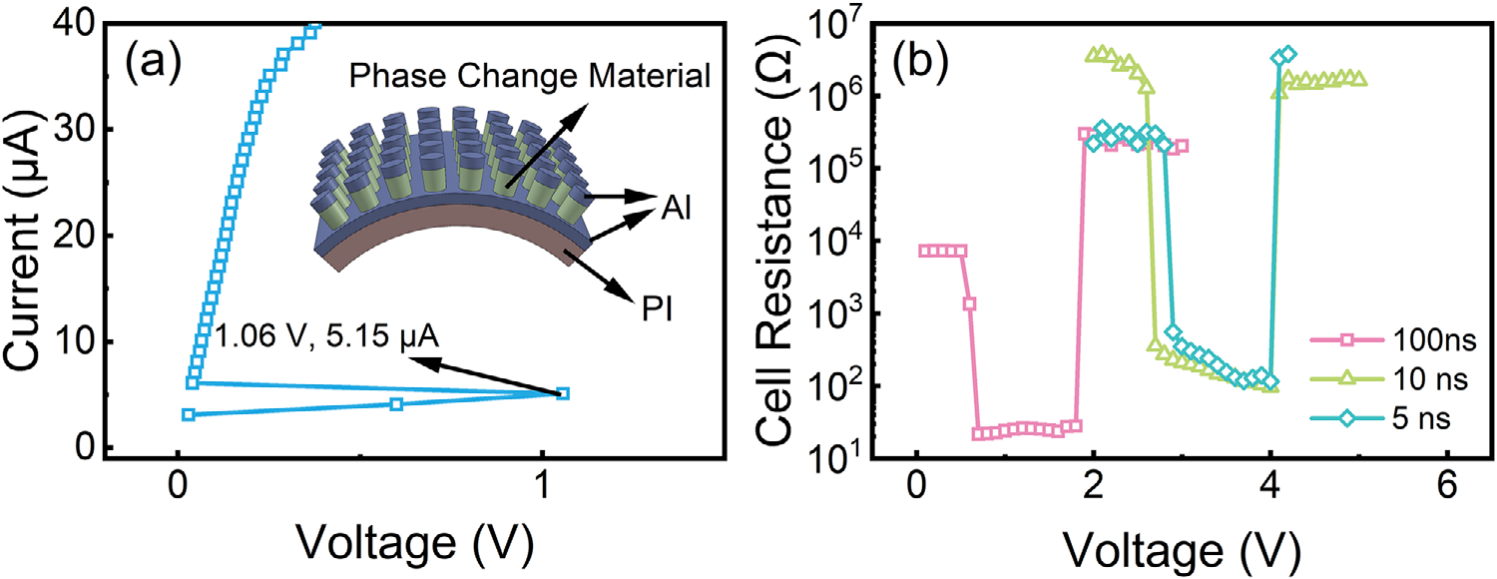
a) *I–V* and b) *R–V* curves of PCM devices based on TiGT films after 1000k bending cycles.

**Figure** **8** demonstrates the electrical characteristics of PCM device based on TiGT films, which was prepared on flexible PI substrates. The inset in Figure 8a) illustrates a schematic of the internal structure of the PCM devices. The total thickness of TiGT films in PCM devices was set to 200 nm, and Al was chosen as the top/bottom electrodes. Figure 8a depicts the *SET* operation in the DC sweep mode. As the scan current increases, the voltage returns to a small value suddenly, displaying a negative phenomenon and implying the phase transition from the amorphous to the polycrystalline state of TiGT film.^[^ ^54^ ^]^ It can be seen from Figure 8a that the threshold (*V_th_*) and threshold current (*I_th_*) for the phase transition are 1.06 V and 5.15 µA, respectively. After 1000k bending cycles, the PCM devices can still exhibit a significant negative resistance process. As shown in Figure 8b, the PCM device based on TiGT films can realize the reversible *SET*/*RESET* switching after 1000k bending cycles at the electronical pulse width of 100 ns, even at 10 and 5 ns, respectively, exhibiting the ultra‐high switching speed. In addition, the discrepancy between high and low resistance exceeds more than two orders of magnitude, ensuring the great erase/write ability of the device. Therefore, the PCM devices based on TiGT films with PI flexible substrates show great potential for application in the field of flexible electronics.

**Figure 9:**
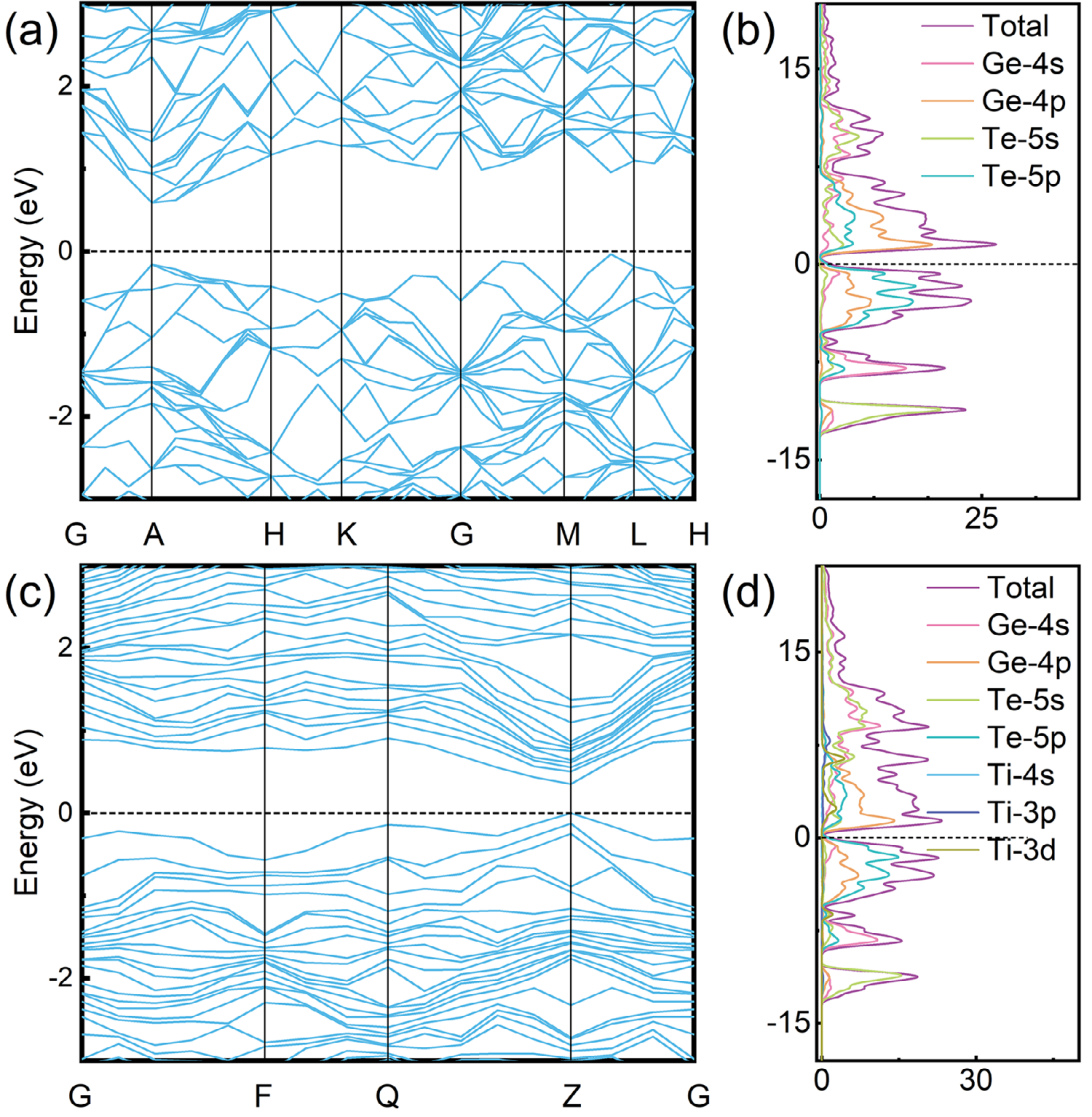
Band structure and PDOS of GT a,b) and TiGT c,d), respectively. The Fermi energy was set to 0 eV.

To understand the evolution of band structure triggered by Ti doping, the band structure and PDOS of GT and TiGT materials were calculated along a commonly used path in the Brillouin zone. As can be seen in Figure 9a, the bottom of conduction band (CB) of intrinsic GT is at point A, while the top of valence band (VB) is between the points M and L, indicating that GT is an indirect bandgap semiconductor. The energy bands in VB have a large broadening, which suggests that the orbital electrons of Ge and Te are shared more obviously. In contrast to the PDOS patterns in Figure 9b, the electronic structure of intrinsic GT is mainly contributed by *p* electrons, forming *p‐p* hybridization interactions. The band gap of GT calculated by PBE functional without spin‐orbit coupling (SOC) is 0.625 eV, which is slightly lower than the experimental value of 0.67 eV.^[^ ^24^ ^]^ Such a discrepancy may be due to the typical underestimation of the band gap in DFT calculations. Although the electron contributions to the electronic structure of GeTe are inconsistent across orbitals, a relatively consistent trend is maintained,^[^ ^55^ ^]^ which suggests a significant hybridization effect between the individual orbitals. Strong *s‐p* covalent bonding can be observed in the density peaks of the *s* and *p* electrons,^[^ ^56^ ^]^ which directly affects the insulator behavior of GT. However, the extra *p* electrons as active conducting carriers are attributed to the energy band near the Fermi energy, which may lead to the metallic behavior of GeTe.^[^ ^57^ ^]^ Thus, GeTe exhibits semi‐metallic properties as a sulfur compound semiconductor.

Figure 9c displays the band structure of TiGT, band gap decreases from 0.625 eV of GT material to 0.352 eV. The reduction in the band gap after Ti doping corresponds to the decrease in the conductivity, which coincides with the results of *R*‐*T* in Figure 2. As illustrated in Figure 9c, the bottom of CB and the top of VB of TiGT are both at Z point, indicating that Ti dopant can transform from the indirect bandgap semiconductor to direct type of GT material. The energy levels at the bottom of CB and the top of VB of TiGT are both close to the Fermi energy, and some of the energy levels have the tendency to broaden, which is the reason why the band gap of TiGT has become smaller and conducive to the leaps of the carriers between the top of VB and the bottom of CB. The changes in the PDOS of TiGT are shown in Figure 9d, which is may be caused by the larger distribution of the peaks in the deeper energy levels relative to the distribution on both sides near the Fermi energy. This is mainly due to the stronger distribution of Ti‐3*d* states in the deeper energy levels. Owing to the difference in the electronegativity of each element, some electrons around Ge and Te are transferred to Ti atoms, which makes the hybridization of the atomic orbitals more intense.

**Figure 10:**
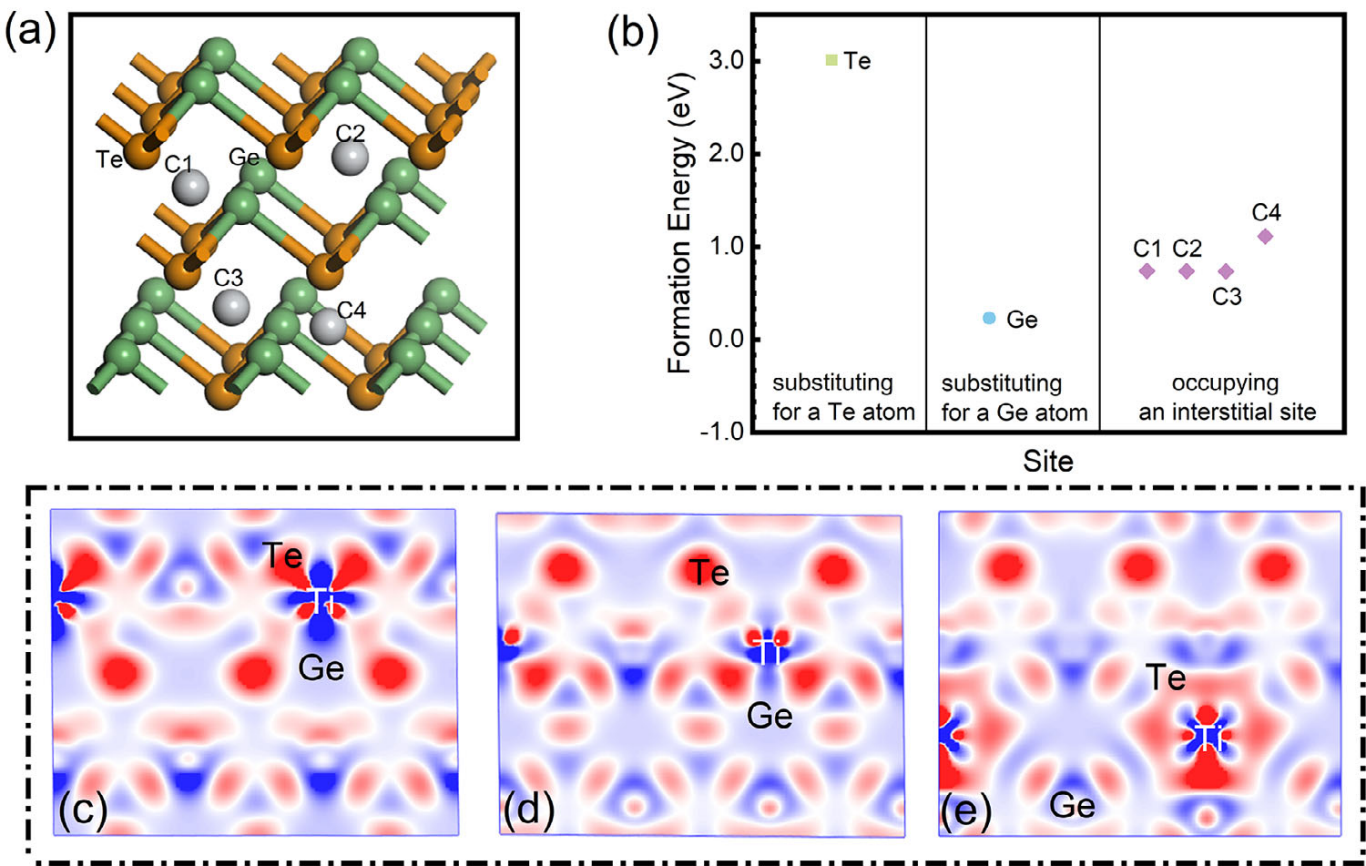
a) Six possible inequivalent doping states for Ti atom in GT: Ge, Te and C*i* (*i* = 1–5). The green, orange, and grey spheres depict Ge, Te, and Ti atoms, respectively. b) Formation energies corresponding to the six different configurations.(c–e) CDDs of Te, Ge and C3. The red and blue electron shadows stand for the accumulation and depletion of electron density, respectively.

To verify the impact of Ti on the bonding state of GT, we calculated the formation energy (*E^f^*) and CDD of GT and TiGT by DFT simulations.^[^ ^58^ ^]^ GeTe (space group R‐3m) was selected as the pristine structure, which is a simple Hexagonal containing 12 Ge and 12 Te atoms. For GeTe, the difference between the experimental lattice constants (*a* = 8.343Å, *c* = 10.668 Å) and the theoretically optimized values (*a* = 8.344Å, *c* = 10.648 Å) is only 0.19%. Figure 10a illustrates the possible inequivalent doping states for a single Ti atom: the Ti atom can occupy either a Ge or Te atom (denoted as Ge, Te), respectively, occupying the C (denoted as C*i* (*i* = 1–5)). The *E^f^* for the different doping positions of Ti in GT are evaluated as^[^ ^59^ ^]^

(4) $$\begin{array}{cc} E^{f} & =E_{tot}\left[Ge_{5-x}Te_{5-y}Ti_{x+y}\right]-E_{tot}\left[Ge_{5}Te_{5}\right]\\ & \;-\left(x+y\right)\mu_{Ti}+x\mu_{Ge}+y\mu_{Te} \end{array}$$

where *E_tot_* [*Ge*_5_ *Te*_5_] and *E_tot_* [*Ge*_5 − *x*_ *Te*_5 − *y*_ *Ti*_*x* + *y*_] are the total energies of pure GT and TiGT. *x+y* denotes the number of Ti atoms. Since there is only one Ti atom, *x* and *y* are only 0 and 1 or vice versa. µ_*Ti*_, µ_*Ge*_, and µ_*Te*_ are the chemical potentials of the corresponding element, which are consistent with chemical potential‐dependent. The calculation consequences of *E^f^* are given in **Figure** **10**b. All the doping types considered have calculated positive formation energies, demonstrating that the insertion of Ti in the crystalline phase is very energetic.^[^ ^60^ ^]^ Among the six different doping configurations, the lowest formation energy is obtained for Ti substituting Ge, and the highest formation energy is calculated after substituting Te, indicating that the dopant Ti atom prefers to occupy the position of Ge to bond with the element Te. The CDD images can reflect the accumulation and consumption of the electrons around the atoms, determining the bonding characteristics and interactions between atoms effectively. In Figure 10c–e, CDD images of Ge, Te and C3 are plotted to analyze the interatomic charges. For intrinsic GT, there is an obvious electron overlap between Ge and Te atoms, emphasizing that Ge and Te atoms exist mainly in the form of covalent bonds with each other. The electron cloud between Ge and Te atoms becomes lighter, which means a decrease of electrons in TiGT. The bright red color of the electrons between the Ti and Te atoms indicates that there is a large concentration of electrons between the two atoms and can explain the relatively strong bonding between the Ti and Te atoms. The results of CDD images show that no matter what position the Ti atom occupies, it can always form a strong covalent bond with the nearby Te atom, corresponding to the analysis of XPS in Figure 5f.

## 3 Conclusion

In summary, the effects of mechanical bending on the thermal stability, chemical bonding, phase structure, microstructure and surface morphology of GT and TiGT films are systematically investigated. The experimental analysis found that the crystallization temperature always maintains at 200–210 °C and 250–265 °C, and the resistance drift coefficients keeps at 10^−1^ to 10^−2^ and 10^−2^ to 10^−3^, respectively, when GT and TiGT films bending with different cycles. Especially, the grain sizes retain without large change, and the film surface shows no signs of fissures or flaws. These results prove that mechanical bending does not affect the thermal stability, surface morphology and crystalline structure of TiGT films. The films exhibit remarkable improvement in thermal stability and dependability after Ti doping, including a considerable rise in the crystallization temperature, a reduction in structural relaxation and a refinement of grain size. PCM devices based on TiGT film were prepared on flexible PI substrates and the complete *SET*/*RESET* operation with a minimum pulse width of 5 ns was confirmed. The calculation results of band structure show that Ti doping can decrease the bandgap from 0.625 eV of GT material to 0.352 eV, exhibiting the direct gap semiconductor. The *E^f^* and CDD results show that the electron cloud heavily aggregates between Ti and Te atoms, and the dopant Ti is inclined to form stronger covalent bonds with Te. All the analysis suggest that TiGT has excellent thermal stability, low resistance drift and superior bending characteristic for the future flexible electronic devices.

## 4 Experimental Section

### Samples and Devices Preparation

The pure GeTe (GT) and Ti_1.3_(GeTe)_98.7_ (TiGT) with the thickness of 200 nm were prepared on PI by sputtering the high purity Ti (99.9999%) and GeTe (99.9999%) target at room temperature. The size of samples was set at 4 cm × 1 cm in length and width, respectively. The deposition rates of monolayer GT and TiGT were predetermined by field emission scanning electron microscopy (FESEM). All deposition processes were in a high‐purity Ar atmosphere with a flow rate of 30 SCCM. The thickness and homogeneity were controlled by the sputtering time and guaranteed by the uniform rotation of substrate holder, respectively. The sputtering pressure and the background vacuum were maintained at 4 × 10^−1^ and 3 × 10^−4 ^Pa, respectively. The voltage of AC target was set to 30 W and the cooling water circulated continuously during the sputtering process.

The prepared samples were bent at different cycles and radius by a homemade mechanical bending apparatus. To prevent the unwanted oxidation of thin films during long‐term bending, a thin SiO_2_ layer with thickness of 5 nm was covered as an antioxidant layer on GT and TiGT samples.

To evaluate the electrical property of device, the PCM cells were prepared on the flexible PI substrates. Al material was selected as the bottom electrode, which was prepared by magnetron sputtering with the thickness of 200 nm. Then, a mask plate was covered on the Al bottom electrode, which was cleaned by ultrasonic cleaning machine. After that, the phase change layer TiGT with a diameter of 10 µm and a height of 200 nm was sputtered on the bottom electrode. Finally, the Al material was deposited on the phase change layer, which was utilized as the top electrode.

### Thermal and Electrical Property

The temperature dependence of resistance (*R–T*) and the resistance drift coefficients of GT and TiGT films with a thickness of 200 nm were measured by using a resistance‐temperature test system, consisting of the high‐resistance, hot/cold stage and customized software based on LabVIEW. The ramp‐up rate of temperature controller was set to 60 °C min^−1^.

The electrical characteristics of PCM devices, including current–voltage (*I–V*) and resistance–voltage (*R–V*), were measured using an arbitrary waveform generator and a semiconductor device analyzer.

### Microscopic Characterization

The phase structure of GT and TiGT films was investigated by X‐ray diffractometry (XRD), which were performed at 40 kV and 30 mA, respectively. The scanning speed and range were set to 5 ° min^−1^ and 20 – 60 °, respectively. The effect of Ti dopant on the bonding properties of GT film was carried out by X‐ray photoelectron spectroscopy (XPS). The surface morphology of GT and TiGT films under different bending cycles was observed by scanning electron microscopy (SEM) and atomic force microscopy (AFM) successively. The thickness of the samples for XRD, XPS, SEM and AFM was set to 200 nm.

The microstructure and element distribution were analyzed by transmission electron microscope (TEM), and the samples with 30 nm thickness were fabricated on the copper mesh with carbon film.

### Theoretical Method and Model

The theoretical calculations were carried out with CASTEP. The band structure and PDOS of GT and TiGT were calculated based on the first principles. The distribution of interatomic charges was analyzed by utilizing charge density difference (CDD) plots. The interactions between ions and electrons were characterized by the projector‐augmented wave (PAW) method. Since van der Waals (vdW) interactions play an active role in the GeTe system, a generalized gradient approximation (GGA) with vdW correction was used to take into account the exchange‐correlation (XC) functionals, where the GGA is described using the Perdew‐Burke‐Ernzerhof (PBE) functional and the vdW interaction using the Becke‐Johnson damping DFT method. The Brillouin zone integrals were performed on well‐converged Monkhorst–Pack k‐point grid. Extensive tests demonstrated that a plane‐wave energy cutoff of 545 eV was adequate to ensure convergence. All the investigated structures were fully relaxed and optimized until the total force on each ion was less than 0.01 eV Å^−1^. At the same time, we modeled the GT crystals based on the XRD phase detection results, and then, the molecular dynamics calculation of the crystal cell was performed through the dynamics calculation task in the Forcite calculation module of Materials Studio software. In the canonical ensemble (NVT), the integral steps of temperature and dynamics were set to 573 K and 1 fs, respectively. 573 K corresponds to the annealing temperature of the film at 300 °C in XRD. The total simulation is 10 ns, and the trajectory information is recorded every 10000 steps. The temperature control algorithm utilized the Nose–Hoover method.

## Conflict of Interest

The authors declare no conflict of interest.

## Data Availability

The data that support the findings of this study are available from the corresponding author upon reasonable request.
